# Clusters formation and fragmentation of nitromethane at 266 nm

**DOI:** 10.1016/j.mex.2020.100909

**Published:** 2020-05-01

**Authors:** D. Martínez, A. Guerrero, E. Prieto, I. Álvarez, C. Cisneros

**Affiliations:** Institute of Physical Science- UNAM, Av. Universidad s/n Chamilpa, 62210, Cuernavaca, Morelos, México

**Keywords:** TOF Mass Spectrometry, Multiphoton Absorption, Clusters

## Abstract

We carry out experiments on the fragmentation of nitromethane by multiphoton absorption at the wavelength 266 nm. This was conducted in a reflectron (Jordan), modified in the laboratory. Due to the large number of fragments, special care has been taken into the calibration of the system, in the simultaneity between the laser pulse and the sample, and the associated electronics to ensure that produced fragment spectra arise from the interaction laser-sample. We emphasize the next aspects of the method:

• Simple design for introducing a gas sample at laser interaction region to facilitate the cluster formation
• Astonishing number of fragments produced by multiphoton absorption.

## Graphical abstract

**Figure:**
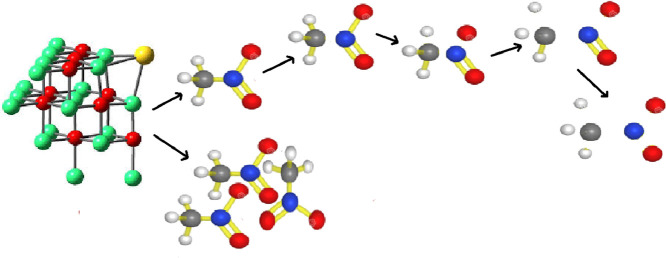

Specifications table

**Table:** 

Subject Area *Physics and Astronomy*	*Select one of the following subject areas:* • Agricultural and Biological Sciences • Biochemistry, Genetics and Molecular Biology • Chemical Engineering • Chemistry • Computer Science • Earth and Planetary Sciences • Energy • Engineering • Environmental Science • Immunology and Microbiology • Materials Science • Mathematics • Medicine and Dentistry • Neuroscience • Pharmacology, Toxicology and Pharmaceutical Science • Physics and Astronomy • Psychology • Social Sciences • Veterinary Science and Veterinary Medicine
More specific subject area: Molecular Physics	*Describe narrower subject area*
Method name: Molecular Multiphoton Absorption and TOF Spectroscopy	*Please specify a name of the method that you customized.* *The method name should be a word or short phrase to describe the methods used in your paper*
Name and reference of original method Time‐of‐Flight Mass Spectrometer with Improved Resolution W. C. Wiley*and*I. H. McLaren. Rev. Sci. Instrum. 26, 1150 (1955); https://doi.org/10.1063/1.1715212	*If applicable, include full bibliographic details of the main reference(s) describing the original method from which the new method was derived.*
Resource availability	*If applicable, include links to resources necessary to reproduce the method (e.g. data, software, hardware, reagent)*

## Method details

We used a pulsed valve and a skimmer to generate a supersonic molecular beam in a collision free system. Between the valve and the skimmer there is an 10 cm stainless steel extension with a 2 mm conical termination inside the chamber, that allowed the adiabatic gas to expand closer to the skimmer. That extension allows cluster formation [1]. There is a 10 mm gap between the end of the extension and the entrance of the skimmer.

The laser radiation (with a Gaussian profile and vertically polarized) was focused into the interaction region using a 15 cm focal length lens. The diameter at the focal point was 80.0 µm. Radiation intensities between 10^9^ and 10^10^ W·cm^−2^ were achieved under these experimental conditions.

A sample of liquid nitromethane from Sigma-Aldrich (purity ~99%) was used. The nitromethane vapor pressure was 3.7 kPa (27.75 torr) at 20°C. The sample was heated at a constant temperature of 28°C and introduced by a pulse valve into the ionization chamber in the gas phase.

The nitromethane gas and laser pulse interact at 90° . The interaction region was located between two polarized electrodes separated by 6 mm with a circular mesh of 90% transparency and a 1 cm diameter. The positive fragments formed at the nanosecond laser pulse were accelerated to the free field region of the R-TOF mass spectrometer by applying an extraction voltage to the electrodes, in the present case it was 1.5 keV [2].

After the ions passed the drift zone, they were directed to the detector, which was a 25 mm dual microchannel plate (Chevron). The signals from the microchannel plate were routed to a fast preamplifier VT 120 (ORTEC) and a picosecond time analyzer (Mod. 9308 ORTEC). A computer (software PTA-32 ORTEC) collected and processed the data according to the arrival time of the mass/charge fragments. The operating pressure was 2 × 10^−6^ torr with an open valve (175 µs) and a base pressure of 10^−8^ torr by the operation of two turbo molecular pumps (Pfeiffer Vacuum and Agilent) while the ions were produced. Once the fragment spectra were obtained it is necessary to identify the correspondent peak to a specific fragment.

The electronics of the detection system allows practically any ions to be recorded, since it is the time difference that differentiate the masses. In the multichannel system, the temporal scan can be done from very short time (400 ns) to very long time (2.7 years) using around 65 thousand channels. The detector is a microchannel plate (MCP), which receives the signal of ions, when hits the detector surface and the small electric charge which is produced goes to the preamplifier and converted to a voltage pulse. The response is of the order of nanoseconds.

The time interval for that single charge mases [1,2] will be: Δ(1,2) = 0.95 × 10^−6^ seg

The difference in time of arrival to the detector for ions with 1 amu and 2 amu is ~ 10 ^- 6^ seg and the detection time is 10^−9^ seconds. Microchannel plate is therefore able to differentiate the times of arrival of each ion. On the other hand, the electronic response time is 10^−12^ seconds. So, an excellent resolution to transform the current generated by the ions to analog and finally digital signal is guaranteed.

**Fig. 1:**
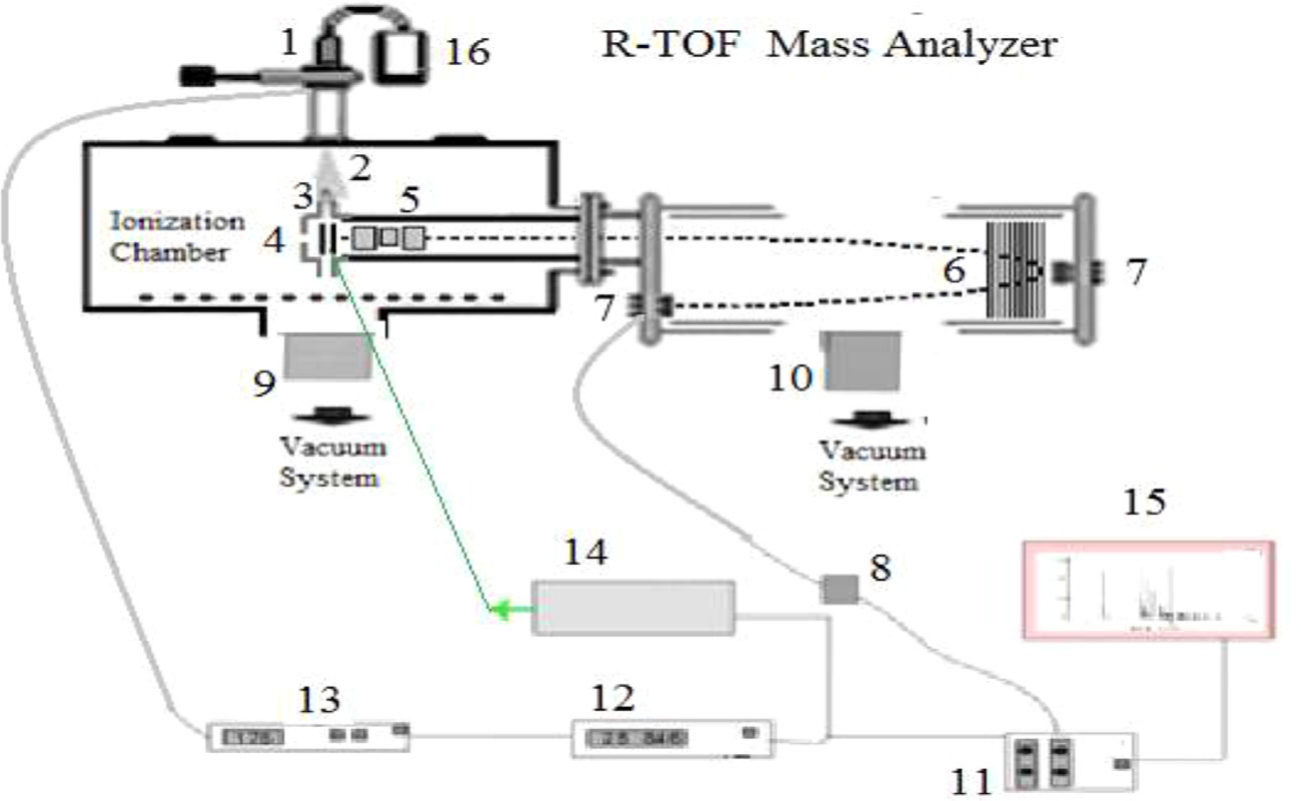
Diagram of the experimental system. 1.Pulsed valve. 2.Extension. 3.Skimmer. 4.Interaction region: polarized electrodes 5. Electrostatic focus & deflection plates 6. Reflector discs, 7. Detector: microchannel plate of the R-TOF 8. Preamplifier. 9 &10. Vacuum system, 11. ORTEC picoameter, 12. Electronic for the control the time for the valve and the laser. 13 Control of valve 14 laser, 15. Data processing. 16. Sample container.

The Fig. 1 shows the experimental setup with the related electronics.

## Declaration of Competing Interest

All authors have participated in the conception and design and writing of the article, and have no affiliation with any organization with a direct or indirect financial interest in the subject matter discussed in the manuscript.

## Appendix Supplementary materials

Image, application 1
