# Supplementary material for: Clusters formation and fragmentation of nitromethane at 266 nm
Source: MethodsX. 2020 May 1;7:100909. doi: 10.1016/j.mex.2020.100909 (PMC7240713; doi:10.1016/j.mex.2020.100909)
Supplement: Supplementary file 1 [file mmc1.docx]

METHOD X

ABSTRACT

We carry out experiments on the fragmentation of nitromethane by multiphoton absorption at the wavelength 266 nm. This was conducted in a reflectron (Jordan), modified in the laboratory. Due to the large number of fragments, special care has been taken into the calibration of the system, in the simultaneity between the laser pulse and the sample, and the associated electronics to ensure that produced fragment spectra arise from the interaction laser-sample. We emphasize the next aspects of the method:

* Simple design for introducing a gas sample at laser interaction region to facilitate the cluster formation

* Astonishing number of fragments produced by multiphoton absorption.

**THE METHOD**

The method of time-of-flight spectrometry has evolved since its practical implementation by Wiley and McLaren in 1955(1), introducing new sources of ionization, improving the resolution and using the new generations of more sensitive detectors.

The photofragments spectra of nitromethane multiphoton absorption were obtained from a high-resolution time of flight mass spectrometer, reflectron (R-TOF) (Fig.6). It is a commercial spectrometer (Jordan TOF Products Inc) modified in the laboratory coupled to a vacuum chamber with a 60 cm diameter, housing the interaction zone where are generated the fragmented ions to be analyzed according to their mass to charge ratio (m/z). Basic design and components are shown in the diagram of the TOF (Fig. 1). As it can be observed for example, the two step source warrant that the mainstream of the fragment ions are accelerated at the same kinetic energy from the source. The uncertainty originated from the spatial distribution of the molecules from the skimmer to the space of the polarized electrodes is minimized from the second electrode before the ions enter into the drift region. Since the trajectory is fixed, we can measure the velocity of the fragmented charged ions. In addition, we are able to use the system as Reflectron, and use the reflector electronics to compensate small differences in the initial velocities at the exit from the source before entering into the drift region. An important factor in the temporal widening in the spectra comes mainly from the focus on the detector, the voltages applied to the Reflectron must be such that they direct the charged particles to the detector correctly. Although the constructor suggests as guide to apply a voltage of +500 volts between the initial and final electrodes of the reflectron and that the reflection voltage is given by the formula V_Ref_=1/2(Vr+Va)+350 where V_Ref_ is the reflectron voltage and Vr, Va the repulsion and extraction voltages respectively, the correct values for each particular situation are found experimentally.

A higher acceleration voltage implies a greater spatial separation between the ions that are directed to the detector. Only a small difference in these voltages results in a notable difference in the ion velocities and consequently in the arrival times to the detector. Although this difference is small increases as the mass increases and the electronics are able to differentiate them (Fig. 1)


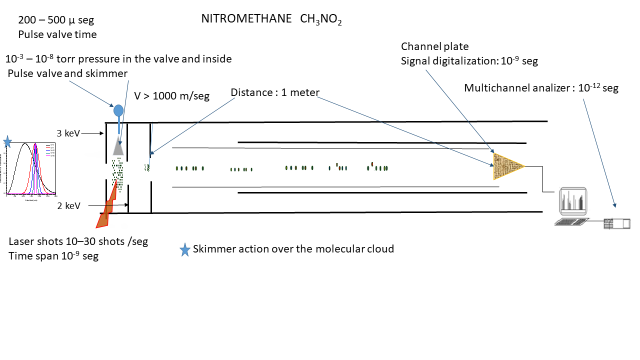
Fig. 1

Velocities at 1 keV: 1 amu: H^+^ : 4.38 x 10^7^ cm/seg

2 amu: H_2_^+^: 3.09 x 10^7^ cm/seg Δ (1,2) = 1.29 x 10^7^ cm/seg

Velocities at 1.5 keV : 1 amu: H^+^ : 5.36 x ~~10 x~~ 10^7^ cm/seg

2 amu: H_2_^+^ : 3.79 x 10^7^ cm/ seg Δ (1,2) = 1.57 x 10^7^ cm/seg

323 amu: (HM)_5_H_2_O^+^ : 2.98 x 10^6^ cm/ seg

367 amu (HM)_6_H^+^ : 2.8 x 10^6^ cm/seg Δ (323,367) = 0.18 x 10^6^ cm/seg

**The electronics**: The electronics are fast response, from the detector (channel plate) whose response is of the order of 10^-9^ sec, preamplifier less than 1 ns, and the multichannel analyzer that manages to differentiate signals up to 10^-12^ sec. This guarantees adequate mass differentiation given the diference in response in orders of magnitude.

**The ion source**: we used a pulsed valve and a skimmer to generate a supersonic molecular beam in a collision free system. Between the valve and the skimmer there is an extension with a conical termination, inside the chamber, that allowed the adiabatic gas to expand closer to the skimmer. There is a 10 mm gap between the end of the extension and the entrance of the skimmer. The geometry of the instrument is such that the molecular beam that leaves the skimmer falls just in half between the extraction and acceleration plates. As can be seen in the inset of the figure 1, the maximum density of the supersonic beam is in the central part. It is in this region where the incident laser is focused, that for 266 nm wavelength has a diameter in the focus of 80 µm and this ensures the greatest spatial coherence between the molecular and laser beams. The synchrony between both beams is described in more detail in the following paragraphs.

**The laser:** The radiation source, a 30 Hz Nd: YAG laser from Spectra Physics was used to generate pulses of 8.5 ns at wavelength of 266 nm. The manufacturer sets the pulse duration. The laser radiation (with a Gaussian profile and vertically polarized) was focused into the interaction region using a 15 cm focal length lens. The diameter at the focal point was 80.0 μm. Radiation intensities between 10^9^ and 10^10^ W·cm^−2^ were achieved under these experimental conditions. To reach the adequate interaction laser/molecule it was necessary to synchronize the time between the laser shot and sample which requires more time to arrive at the center of the interaction region.

The synchronization was attained opening the valve in advance to the emission of the laser pulse, this takes into consideration the time of arrival of the molecular cloud at the outlet of the valve towards the center of the region of interaction with the laser beam. The pulse valve can be controlled manually to obtain the maximum signal, however the best way to synchronize the laser shot and the sample plume at the center of the interaction region is to use a special electronics that we designed for that propose. When the laser generates a pulse of light, the control electronics provides a pulse of voltage TTL and with a retarding electronic system can simultaneously generate a pulse delay with respect to the first. This pulse is used to generate a variable voltage, that is used to activate the valve. If t_d_ is the time of the open valve to obtain the optimal synchrony between laser pulse-molecular sample, then its value: t_d_ = (1/ʋ) – t_1_ ; ʋ is the laser frequency and t_1_ delayed time compared to the TTL previous signal. See Fig.2. A poor synchronization between the firing of the laser and the opening of the valve causes unused laser pulses which leads to a shorter life time in the excitation lamps, in addition to obtaining a bad signal in the detector.


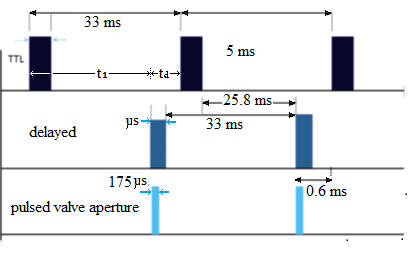


Fig. 2. Diagram of the synchronization of the TTL pulses, delayed and opening of the valve

**The sample**

In order to test the present experimental arrangement Nitrometane was studied . The study of Nitromethane was chosen due to its great interest in atmospheric and interstellar chemistry as well as being an efficient energy source. It is a complex molecule that has been studied with different experimental techniques, in particular the one used in the present work is the multiphotonic ionization / dissociation induced by laser absorption at 266 nm wavelength. Multiphoton spectroscopy is an excellent method since it is possible to access high-energy molecular states being able to analyze the dissociation and ionization of large molecules.

A sample of liquid nitromethane was purchased from Sigma-Aldrich (purity ~99%). The nitromethane vapor pressure was 3.7 kPa (27.75 torr) at 20°C. The sample was heated at a constant temperature of 28°C and introduced by a pulse valve into the ionization chamber in the gas phase. The pulsed valve had an extension with a conical termination inside the chamber that allowed the gas adiabatically to expand closer to the skimmer to generate a supersonic molecular beam in a collision-free system. Being a supersonic beam velocity (v ~ 10^4^ cm/s) and considering the distance traveled of ~ 15 cm from valve to center of the interaction region, the time in advance of the opening should be of the order of some milliseconds. The final tuning is adjusted manually until the best signal is obtained.

The nitromethane gas and laser pulse interact at 90° (Fig. 3). This region was located between two polarized electrodes separated by 6 mm. Both have a circular mesh of 90% transparency and a 1 cm diameter. The positive fragments formed by the nanosecond laser pulse were accelerated to the free field region of the R-TOF mass spectrometer by applying an extraction voltage to the electrodes, in the present case it was 1.5 keV.

**
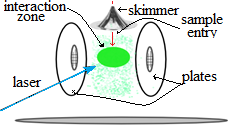
**

Fig.3 Laser-sample interaction

Finally, after the ions passed the drift zone, they were directed to the detector, which was a dual microchannel plate (Chevron). The signals from the microchannel plate were routed to a fast preamplifier VT 120 and a picosecond time analyzer (ORTEC). A computer collected and processed the data according to the arrival time of the mass/charge fragments. The operating pressure was 2 × 10^−6^ torr with an open valve (175 µs) and a base pressure of 10^-8^ torr by the operation of two turbo molecular pumps (Pfeiffer Vacuum and Agilent) while the ions were produced. Once the fragment spectra were obtained it is necessary to identify the correspondent peak to a specific fragment. To do this it is needed to calibrate the system. In order to achieve that, when the molecular sample arrives to the center of the ion source and is ionized from the laser, the charged ions feel the extraction voltage and the cation fragments are accelerated toward the drift region. The gained kinetic energy will be proportional to the potential difference (V) between the electrodes: ½ mv^2^ = qV and v = (2qV/m)^1/2^ and since v = L/t were L is the drift region length and t is the flight time: t = L (m/2qV)^1/2^, the difference transit time for masses m_1_ and m_2_ can be expressed as follows: Δt = ( L m_1_^1/2^ – L m_2_^1/2^) / (2qV)^1/2^

For two ions with the same charge and kinetic energy, but different mass m_1_v_1_^2^ = m_2_v_2_^2^, if L is the drift length, we have t_1_/m_1_^1/2^  = t_2_/m_2_^1/2^ , and knowing the time for one fragment, it is possible to convert the time of flight for the other fragments to a mass spectrum.

As an example, the spectrum of the nitromethane fragments obtained in the interaction with laser Nd: YAG at 266 nm and 4.5 ns is shown (Fig.4 ). The Table 1 shows some of the fragments identified using the method described and followed a table with the clusters identified.


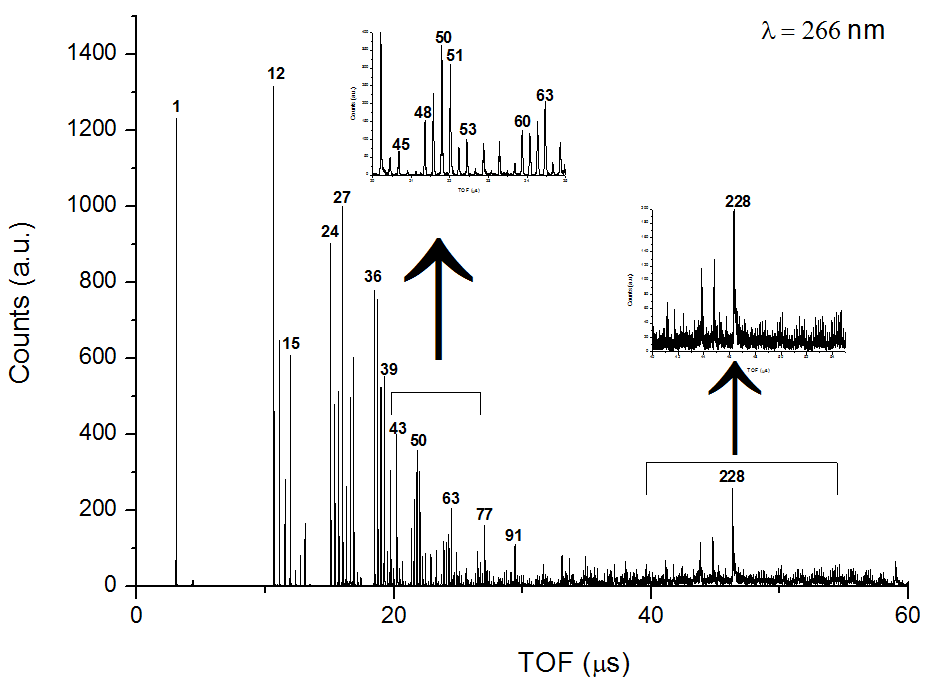


Fig.4 flight time spectrum of nitromethane at 266 nm.

Table I.

| ION | m/z | ION | m/z | ION | m/z | ION | m/z |
| --- | --- | --- | --- | --- | --- | --- | --- |
| H^+^ | 1 | C_2_^+^ | 24 | C_3_^+^ | 36 | CO_2_ H^+^ - CH_3_NO^+^ | 45 |
| H_2_^+^ | 2 | C_2_H^+^ | 25 | C_3_H^+^ | 37 | CO_2_ H_2_^+^ - NO_2_^+^ | 46 |
| C^+^ | 12 | C_2_H_2_^+^ | 26 | C_3_H_2_^+^ | 38 | C_2_HNO^+^ | 55 |
| CH^+^ | 13 | C_2_H_3_^+^ | 27 | C_3_H_3_^+^ | 39 | C_2_H_2_NO^+^ - C_2_O_2_^+^ | 56 |
| CH_2_^+^ | 14 | CNH_2_^+^ - CO^+^ | 28 | C_2_O^+^ | 40 | C_2_H_3_NO^+^ - C_2_O_2_H^+^ | 57 |
| CH_3_^+^ | 15 | CNH_3_^+^-HCO^+^ | 29 | C_2_HO^+^ | 41 | CNO_2_^+^ | 58 |
| O^+^- CH_4_^+^ | 16 | NO^+^ - H_2_CO^·+^ | 30 | CNO^+^  - C_2_H_2_O^+^ | 42 | CHNO_2_^+^ | 59 |
| OH^+^- CH_5_^+^ | 17 | COH_3_^+^ - NOH^+^ | 31 | CHNO^+^  - C_2_H_3_O^+^ | 43 | CH_2_NO_2_^+^ | 60 |
| H_2_O^+^- CH_6_^+^ | 18 | NOH_2_^+^ | 32 | CH_2_NO^+^ | 44 | CH_3_NO_2_^+^ | 61 |

Clusters

| Cluster | m/z | Cluster | m/z |
| --- | --- | --- | --- |
| (NM)H | 62^*^ | (NM)_2_[C_2_H_3_NO-C_2_O_2_H] | 179 |
| (NM)H_2_ | 63 | (NM)_3_ | 183^*^ |
| (NM)CH | 74 | (NM)_3_CH_3_ | 198^*^ |
| (NM)O | 77 | (NM)_3_[CNH_3_-HCO] | 212 |
| (NM)NO | 91^*^ | (NM)_3_CH_3_NO | 228^*^ |
| (NM)_2_NO | 152^*^ | (NM)_5_H_2_O | 323 |
| (NM)_2_O_2_H | 155^*^ | (NM)_6_H | 367^*^ |

The asterisks indicate clusters also identified by Ferreira^4^.

**Calibration**

With the calculated m / z value and flight times, a calibration curve is constructed from which the equation that conforms to the values of the masses is obtained. The Figure 5 shows the calibration curve for nitromethane at 266 nm and the related equation.


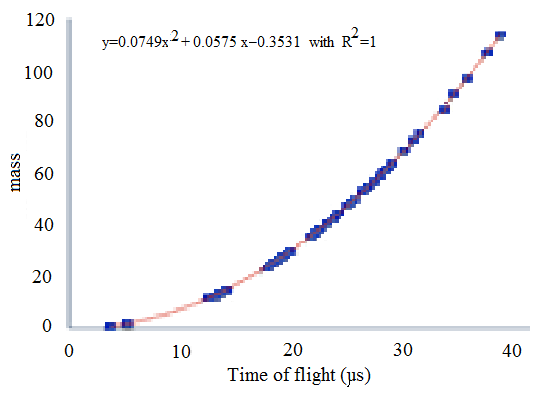


Figure 5. Calibration curve for nitromethane ions formed by multi-photon absorption at 266 nm

It is interesting to notice that the spectra in the figures 4,5 show fragments with masses greater than 61, which corresponds to the mass of the molecular ion. In the Table I of identified ions, there are fragments containing C_2_ and C_3_. This is due to the presence of clusters. Pulse valve, extension tube and the skimmer favor the formation of aggregates or clusters, which, in turn originate the fragments with C_2_, C_3_, C_2_H_2_, C_3,_ and so on. The presence of clusters and the duration of the laser pulse produce large number of fragments (2,3)

**The electronics for the time of flight**

The important aspect in the TOF experiments are the times involved on the different steps in the processes. The time interval of the laser shot for a 30 Hz will be 30 shots per second. The aperture of the valve, in the range of microseconds (~175 µs). The molecular beam is supersonic (~10^2^ m/seg). The temporal width of the laser is in the nanosecond range 10^-9^ seg. The molecular dissociation in the case of nitromethane is of the order of 10^-12^seg. The electronics of the detection system allows practically any ions to be recorded, since it is the time difference that differentiate the masses. In the multichannel system, the temporal scan can be done from very short time (400 ns) to very long time (2.7 years) using around 65 thousand channels. The detector is a microchannel plate (MCP), which receives the signal of ions, when hits the detector surface and the small electric charge which is produced goes to the preamplifier and converted to a voltage pulse. The response is of the order of nanoseconds. Figure 6 shows the electronic configuration of the experimental system


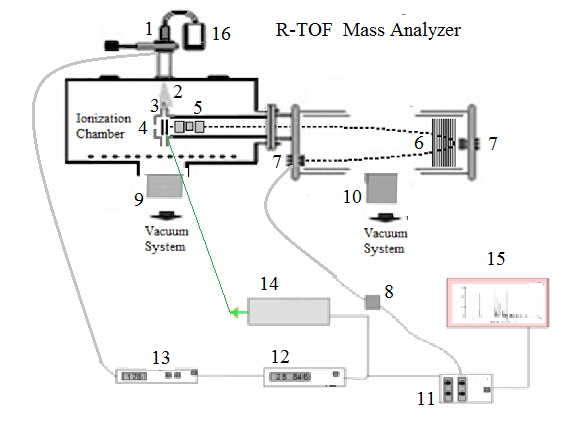


Fig. 6. Diagram of the experimental system. 1.Pulsed valve. 2.Extension. 3.Skimmer. 4.Interaction region: polarized electrodes 5. Electrostatic focus & deflection plates 6. Reflector discs, 7. Detector: microchannel plate of the R-TOF 8. Preamplifier. 9 &10. Vacuum system, 11. ORTEC picoameter, 12. Electronic for the control the time for the valve and the laser. 13. Control of valve 14. laser, 15. Data processing. 16. Sample container

**The resolution**

Considering the velocities for the single charge fragments with amu 1 and amu 2 at 1 keV acceleration potential and the time to travel one meter to the detector, we have:

For 1 amu, v = 4.38 x 10^7^cm/seg the time to travel 1m is: t = 2.28 x 10^-6^seg

For 2 amu v = 3.09 x 10^7^cm/seg the time to travel 1m is: t = 3.23 x 10^-6^seg

The time interval for that single charge mases (1,2) will be: Δ(1,2) = 0.95 x 10^-6^ seg

The difference in time of arrival to the detector for ions with 1 amu and 2 amu is ~ 10 ^- 6^ seg and the detection time is 10^-9^ seconds. Microchannel plate is therefore able to differentiate the times of arrival of each ion. On the other hand, the electronic response time is 10^-12^ seconds. So, an excellent resolution to transform the current generated by the ions to analog and finally digital signal is guaranteed.

To show this consider the difference in transit time Δt for two mases m1 and m2, it will be:

Δt = ( Lm_1_^1/2^ – Lm_2_^1/2^ )/ (2qV)^1/2^ ,

This can be approximated [ (Δt)^2^->0] by

m/Δm = t/2Δt

where t corresponds to the time of flight of the selected ion and Δt corresponds to the width of the signal at half the maximum (FWHM). The Figure 7 shows some of the resolution values calculated for different masses.


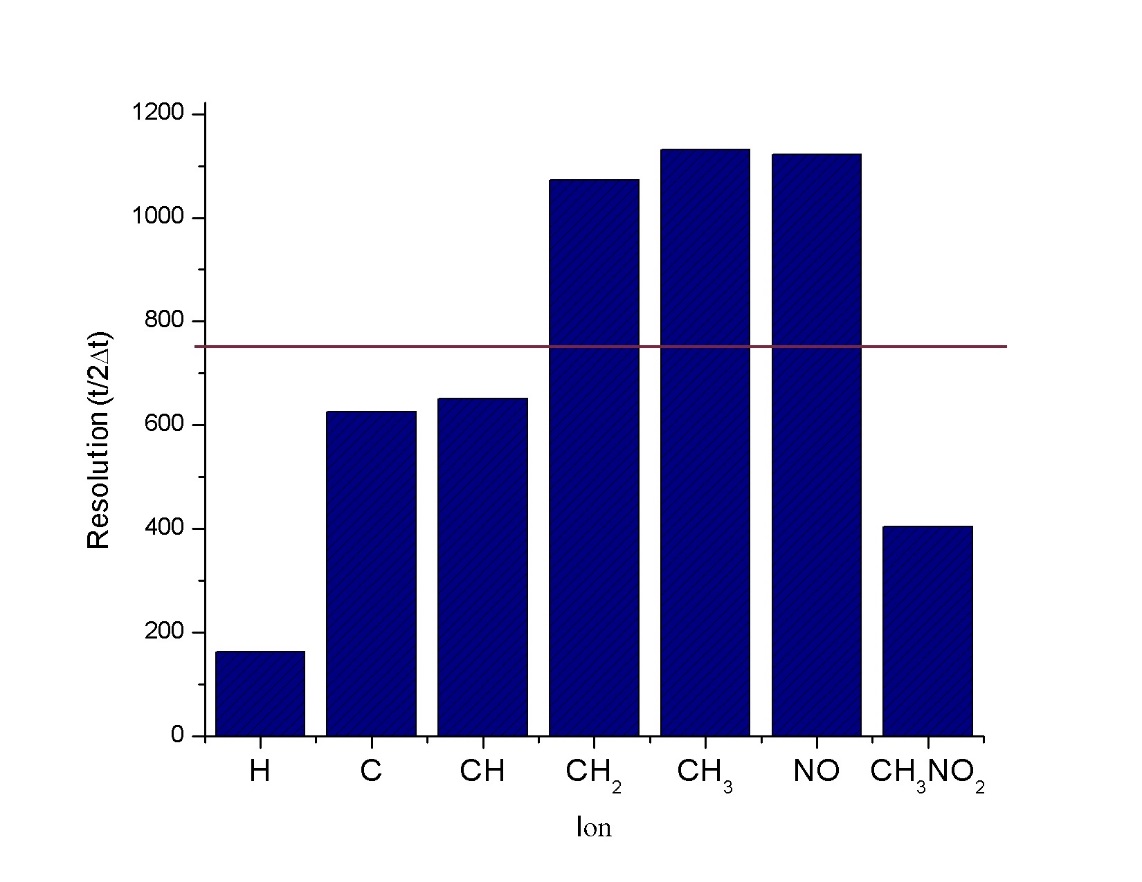


Fig. 7. Resolution for different masses of a TOF spectrum of nitromethane at 266 nm, the horizontal line indicates the average value that is 738.

With the characteristics of the pulse laser (30 Hz), the synchrony between opening of the valve and the pulses of the laser radiation, guarantees that different masses-ionized fragments will be detected without overlap and will be processed equally due to the fast electronics.

**Conclusion.**

Using a R-TOF and the multiphoton absorption technique we are able detect the copious fragmentation of a sample of nitromethane. The experimental design allows the cluster formation and further fragmentation. The experimental parts, as the introduction of the sample, the synchrony of the sample and laser shots, the calibration of the spectrum and the influence in the way of of the introduction of the sample and all the associated electronics are emphasized showing the interest of the present design.

**Declaration of Competing Interest**

All authors have participated in the conception and design and writing of the article, and have no affiliation with any organization with a direct or indirect financial interest in the subject matter discussed in the manuscript.

**Acknowledgments**
This work was supported by DGAPA PAPIIT grants No. IN104019 and No.IN104318

**References**

### 1.- Time‐of‐Flight Mass Spectrometer with Improved Resolution

### [W. C. Wiley](https://aip.scitation.org/author/Wiley%2C+W+C) *and* [I. H. McLaren](https://aip.scitation.org/author/McLaren%2C+I+H)

### Rev. Sci. Instrum. 26, 1150 (1955); <https://doi.org/10.1063/1.1715212>

**2. -** Atomic and Molecular Beam Methods

Giacinto Scoles Editor and Davide Bassi, Udo Buck and Derek Laine Ass.Editors

Chapter 3, section 323. Oxford University Press 1988

**3.-** Novel Reaction Mechanisms Pathways in Electron Induced Decomposition of Solid Nitromethane (CH_3_NO_2_) and D_3_- Nitromethane (CD_3_NO_2_)

Ralf I. Kaiser and Pavlo Maksyutenko

J. Phys. Chem. C 2015, 14653-14668

4.- F. Ferreira da Silva, S. Ptasinska, S. Denifl, D. Gschliesser, J. Postler, C. Matias,T. D. Märk, P. Limão-Vieira and P. Scheier; Electron interaction with nitromethane embedded in helium droplets: Attachment and ionization measurements; [J. Chem. Phys.](https://doi.org/10.1063/1.3656680) **135**, 174504 (2011).
